# “INTERGROWTH21st vs customized fetal growth curves in the assessment of the neonatal nutritional status: a retrospective cohort study of gestational diabetes”

**DOI:** 10.1186/s12884-020-2845-y

**Published:** 2020-03-04

**Authors:** Juan Jesús Fernández-Alba, Estefanía Soto Pazos, Rocío Moreno Cortés, Ángel Vilar Sánchez, Carmen González Macías, María Castillo Lara, Luis Moreno Corral, José Antonio Sainz Bueno

**Affiliations:** 1 Department of Obstetrics and Gynecology, University Hospital of Puerto Real, Ctra. Nacional IV, km. 665. Puerto Real, 11510 Cádiz, Spain; 2 Nursing Department, Faculty of Health Sciences, Cádiz University, Campus de la Asunción, Av. Ana de Viya, 52, Cadiz, 11510 Spain; 3 Maternity Unit, Valme University Hospital, Seville, Spain

**Keywords:** Small for gestational age (INFANT SGA), Fetal macrosomia, Diabetes, Gestational (gestational diabetes), Birth weight, Fetal growth, Fetal malnutrition, Infant overnutrition

## Abstract

**Background:**

Gestational diabetes mellitus is associated with increased incidence of adverse perinatal outcomes including newborns large for gestational age, macrosomia, preeclampsia, polyhydramnios, stillbirth, and neonatal morbidity. Thus, fetal growth should be monitored by ultrasound to assess for fetal overnutrition, and thereby, its clinical consequence, macrosomia. However, it is not clear which reference curve to use to define the limits of normality. Our aim is to determine which method, INTERGROWTH21st or customized curves, better identifies the nutritional status of newborns of diabetic mothers.

**Methods:**

This retrospective cohort study compared the risk of malnutrition in SGA newborns and the risk of overnutrition in LGA newborns using INTERGROWTH21st and customized birth weight references in gestational diabetes. The nutritional status of newborns was assessed using the ponderal index. Additionally, to determine the ability of both methods in the identification of neonatal malnutrition and overnutrition, we calculate sensitivity, specificity, positive predictive value, negative predictive value and likelihood ratios.

**Results:**

Two hundred thirty-one pregnant women with GDM were included in the study. The rate of SGA indentified by INTERGROWTH21st was 4.7% vs 10.7% identified by the customized curves. The rate of LGA identified by INTERGROWTH21st was 25.6% vs 13.2% identified by the customized method. Newborns identified as SGA by the customized method showed a higher risk of malnutrition than those identified as SGA by INTERGROWTH21st. (RR 4.24 vs 2.5). LGA newborns according to the customized method also showed a higher risk of overnutrition than those classified as LGA according to INTERGROWTH21st. (RR 5.26 vs 3.57). In addition, the positive predictive value of the customized method was superior to that of INTERGROWTH21st in the identification of malnutrition (32% vs 27.27%), severe malnutrition (22.73% vs 20%), overnutrition (51.61% vs 32.20%) and severe overnutrition (28.57% vs 14.89%).

**Conclusions:**

In pregnant women with DMG, the ability of customized fetal growth curves to identify newborns with alterations in nutritional status appears to exceed that of INTERGROWTH21st.

## Background

Gestational diabetes mellitus (GDM) is associated with increased incidence of adverse perinatal outcomes including newborns large for gestational age (LGA), macrosomia [1–4], preeclampsia [5], polyhydramnios, stillbirth, and neonatal morbidity [6]. Newborns of mothers with GDM are heavier and have greater skinfold measures and adiposity than offspring of mothers without GDM. Later in life, children of diabetic mothers more frequently develop early overweight or obesity, type 2 diabetes, and metabolic syndrome [7–9].

In pregnant woman with GDM, ultrasound is used to monitor growth as overnutrition is common and influences management. Prenatally, fetal overgrowth is suspected when the ultrasound estimated fetal weight (EFW) is abnormally elevated. An EFW higher than the 90th centile indicates an LGA fetus. In preterm fetuses, this method is more accurate than that based only on the absolute value of the EFW (EFW greater than 4000 or 4500 g) [10]. By considering gestational age at the time of ultrasound, excessive fetal growth can be identified before the term [11].

Traditionally, fetal growth has been evaluated by comparing estimated fetal weight with population-based reference curves. Similarly, recent reports of the INTERGROWTH21st Project recommend using a single standard for fetal growth and birthweight [12–14].

Alternatively, a customized approach that uses a mathematical model of maternal anthropometric variables to predict the optimal weight at term of each fetus has gained strength recently [15, 16]. This optimal weight at term can be combined with a fetal proportionality weight curve to calculate a customized curve for each mother in each pregnancy that can be used to predict birth weight and fetal growth [17].

A few studies have compared INTERGROWTH21st and customized curves ability to identify fetuses at high risk of adverse perinatal outcomes, but not their ability to identify alterations in neonatal nutritional status [17–21]. In this study, we used the ponderal index (PI) to assess the nutritional status of newborns of GDM mothers. We hypothesized that, in pregnant women with GDM, customized curves identify the nutritional status of the newborn more accurately than INTERGROWTH21st. This study aims to determine which method, INTERGROWTH21 or customized curves, better identifies newborns with an abnormal PI, as an indicator of the nutritional status of newborns of diabetic mothers.

## Methods

### Design

This historical cohort study was conducted at the Department of Obstetrics and Gynecology of the University Hospital of Puerto Real (Cádiz/Spain). Medical records of all consecutive singleton births that occurred from January 2016 through March 2018 were retrieved from our database of information prospectively collected. Only pregnant women with GDM were included in the study. Congenital anomalies or stillborn babies were excluded from our study because of possible changes in fetal and birth weights. Gestational age was established based on the last menstruation and first ultrasound (usually at 11–12 weeks). In cases where the gestational age by ultrasound differed by ≥1 week, the last menstruation was corrected and stored in the information system.

Accepting an alpha risk of 0.05 and a beta risk of 0.2 in a one-sided test, 39 exposed subjects (SGA and LGA) and 195 in the non-exposed (AGA) are necessary to recognize as statistically significant a relative risk greater than or equal to 3. A proportion of abnormal PI in the exposed group has been estimated to be 0.1.

An exhaustive explanation of our customized method can be found in the study published by Fernández Alba et al. [22]. This method (based on the one proposed by Gardosi) [16], predicts the weight that the newborn will have at 40 weeks based on fetal sex and maternal variables (age, height and weight at the beginning of pregnancy). Next, the weight at each gestational age is calculated as a proportion of the weight at 40 weeks, according to the proportionality curve proposed by Hadlock et al. [23]

To compare the two identification methods (INTERGROWTH21st and customized), two analyses were performed:

1. Determination of the risk of alterations in the nutritional status of the newborn (malnutrition or overnutrition). To calculate the risk of neonatal malnutrition, the exposed group included newborns classified as small for gestational age (SGA) and the reference group included newborns classified as appropriate for gestational age (AGA). The same analysis was performed twice: one using INTERGROWTH21st as the curve of reference and another using our customized fetal growth curves as the reference. To calculate the risk of neonatal overnutrition and severe neonatal overnutrition, the exposed group included newborns classified as LGA and the reference group included newborns classified as AGA. Again, the analysis was performed twice: once using the INTERGROWTH reference method and the other using our customized curves. To check if there were statistically significant differences, the risks obtained were compared using the method proposed by Altman and Bland [24].
2. Determination of the sensitivity, specificity, positive predictive value (PPV), negative predictive value (NPV), positive likelihood ratio (LR+), negative likelihood ratio (LR-) and receiver operating characteristic (ROC) curves of both methods for identifying the nutritional status of the newborn.
3. Comparison of the ROC curves using the method proposed by deLong [25].

#### Definitions

The diagnosis of GDM was established when at least two of the following four plasma glucose levels (measured at fasting, 1 h, 2 h, and 3 h after a 100 g oral glucose tolerance test) were equal to or greater than 105 mg/dL, 190 mg/dL, 165 mg/dL and 145 mg/dL, respectively.

According to birth weight, newborns were classified as SGA (birthweight <10th centile), AGA (birthweight between 10th and 90th centile), or LGA (birthweight >90th centile) both by INTERGROWTH21st curves and by our customized curves.

The nutritional status of the newborn was evaluated using the ponderal index (PI) of Rohrer [26] adjusting by sex and gestational age. Proposed by Rohrer, the PI indicates how heavy a newborn is relative to its length [27–30]. The formula is as follows: PI = (weight in g × 100)/ (length in cm)^3^. Because the PI relates to weight and length, it indicates body proportions, thus providing information about the nutritional status of newborn [31]. The height of the newborns was measured using a tallimeter of the SECA® brand model 210 with a graduation measuring range of 5 mm.

Neonatal malnutrition was defined as the PI <10th centile; a PI between the 10th percentile and 90th centile was classified as normal; and a PI >90th centile was classified as neonatal overnutrition.

### Statistical analyses

Categorical data were summarized as counts and percentages. The distributions of continuous data were assessed using the Shapiro-Wilk test. Continuous data with a normal distribution were summarized as mean and standard deviation; by contrast, when the data showed a non-normal distribution, we used the median and the interquartile range as a measure of central tendency.

The χ2-test was used to evaluate the differences in the frequency of SGA and LGA newborns according to each classification method.

The risks of malnutrition in newborns classified as SGA, by the INTERGROWTH21st and by our customized method, were calculated. Likewise, the risks of overnutrition in newborns classified as LGA by these two methods were calculated. The results were expressed as relative risk (RR) and 95% confidence interval (95% CI). The RR thus obtained were compared to each other to verify if there were statistically significant differences. To do this, we calculate the difference between the log of RR and the standard error of this difference. Then a test of interaction was obtained obtaining a z-score and its corresponding *p* value [24].

The, PPV, NPV, sensitivity, specificity, LR+ and LR- for the identification of malnutrition and overnutrition were calculated for newborns classified as SGA and LGA by both methods (INTERGROWTH21st and customized curves). Finally, the ROC curves corresponding to each method were prepared and compared with each other using the method proposed by deLong [25].

A *p*-value less than 0.05 was deemed statistically significant. All statistical analyses were performed using R statistical software v. 3.5.2 [32].

## Results

This study recruited 234 pregnant women with GDM. In 3 cases the length of the newborn was missing, so 231 women remained in the study.

The maternal characteristics and perinatal outcomes are displayed in Table 1. The mean PI was 2.68 (SD: 0.26). The results of neonatal nutritional status are shown in Table 2. The incidence of newborns with a PI <10th centile was 8.7%, and the incidence of newborns with a PI >90th centile was 13.9%.

**Table 1:** Maternal characteristics and perinatal outcomes

Variable	Value
**Maternal age (years)**:	34.59 (4.7) *
**Maternal weight at the beginning of pregnancy (kg):**	69 (25) **
**Maternal height (cm):**	162.56 cm (6.13) *
**Maternal BMI at the beginning of gestation (kg/m**^**2**^**):**	25.92 kg/m^2^ (8.62) **
- Underweight (BMI < 18.5)	8 (3.4%) ***
- Normal BMI (18.5–24.9)	94 (40.2%) ***
- Overweight (BMI 25–29.9)	61 (26.1%) ***
- Obesity (BMI ≥ 30)	71 (30.3%) ***
**Hypertensive disorders of pregnancy**	11 (4.8%) ***
- Gestational hypertension	5 (2.2%) ***
- Chronic hypertension	5 (2.2%)
- Preeclampsia	1 (0.4%)
**Management of gestational diabetes**	
glycemic control with diet and exercise	151 (65.4%) ***
Insulin required	80 (34.6%) ***
**Gestational age at birth (weeks):**	39 weeks (2) **
- < 34 weeks	5 (2.1%) ***
- 34–34 + 6 weeks	12 (5.1%) ***
- 37–40 + 6 weeks	198 (84.6%) ***
- ≥ 41 weeks	19 (8.1%) ***
**Neonatal gender**	
- Female	102 (43.6%) ***
- Male	132 (56.4%) ***
**Birth weight (g)**	3302 (506.47) *
**Birth length (cm)**	49.68 cm (2.19) *
**Apgar score at 1 min**	
< 7	8 (3.5%) ***
≥ 7	223 (96.5%) ***
**Apgar score at 5 min**	
< 7	0
≥ 7	231 (100%) ***

*SD* Standard deviation, *IQR* Interquartile range, *BMI* Body Mass Index

* Mean and standard deviation; ** Median and interquartile range; *** Absolute frequency and percentage

**Table 2:** Nutritional status at birth

Nutritional status at birth	N (%)
Malnutrition (PI < 10th centile)	20 (8.7%)
- Non-severe malnutrition (PI between 3rd and 10th centile)	11 (4.8%)
- Severe malnutrition (PI <3rd centile)	9 (3.9%)
Norm nutrition (PI between 10th and 90th centile)	179 (77.5%)
Overnutrition (PI > 90th centile)	32 (13.9%)
- Non-severe overnutrition (PI between 90th and 97th centile)	24 (10.4%)
- Severe overnutrition (PI >97th centile)	8 (3.5%)

*PI* Ponderal index

Table 3 shows the distribution of SGA, AGA and LGA newborns identified by each, INTERGROWTH21st and the customized, method. The rate of SGA newborns identified by INTERGROWTH21st was 4.8% versus 10.8% identified by our customized method. The rate of LGA newborns identified by INTERGROWTH21st was 26% vs 13.4% identified by the customized method (*p* < 0.001).

**Table 3:** Birthweight distribution classified by INTERGROWTH21st and the customized method and proportion of newborns with PI <10th centile, normal PI and PI>90th centile in each of the groups

	INTERGROWTH21st	Customized method
SGA: N (%)	11 (4.8%)	25 (10.8%)
PI <10th centile	3 (27.3% of IG SGA)	8 (32% of CM SGA)
PI between 10th and 90th centile	8 (72.7% of IG SGA)	17 (68% of CM SGA)
PI >90th centile	0	0
AGA: N (%)	160 (69.3%)	175 (75.8%)
PI <10th centile	16 (10% of IG AGA)	12 (6.9% of CM AGA)
PI between 10th and 90th centile	131 (81.9% of IG AGA)	147 (84% of CM AGA)
PI >90th centile	13 (8.1% of IG AGA)	16 (9.1% of CM AGA)
LGA: N (%)	60 (26%)	31 (13.4%)
PI <10th centile	1 (1.7% of IG LGA)	0
PI between 10th and 90th centile	40 (66.7% of IG LGA)	15 (48.4% of CM LGA)
PI >90th centile	19 (31.7% of IG LGA)	16 (51.6% of CM LGA)

*SGA* Small for gestational age, *AGA* Adequate for gestational age, *LGA* Large for gestational age, *PI* Ponderal Index, *IG* INTERGROWTH21st, *CM* Customized method

Most newborns classified as SGA by INTERGROWTH21st also were identified as SGA by the custom method (10 of 11). However, of the 25 newborns classified as SGA by the customized method, only 40% (10) was classified as SGA by INTERGROWTH21st and the remaining 60% was classified as AGA.

Ten of the 11 newborns classified as SGA according to INTERGROWTH21st were classified as SGA and 1 as AGA according to the custom method. In contrast, 48.3% of the 60 newborns classified as LGA according to INTERGROWTH21st were classified as AGA according to the custom method and only 51.7% were classified as LGA.

On the other hand, only 10 (40%) of the 25 newborns classified as SGA by the custom method were also classified as SGA by INTERGROWTH21st and the remaining 60% was classified as AGA. 100% of newborns classified as LGA by the custom method were also classified as LGA by INTERGROWTH21st.

The risk of presenting a PI <10th centile in newborns classified as SGA by customized curves was 4.24 times that of newborns classified as AGA (RR 4.24, 95% CI 1.93–9.33). Using INTERGROWTH21st the newborns classified as SGA had 2.5 times of presenting a PI <10th centile than those classified as AGA but, with this method, the risk was not statistically significant (RR 2.5, 95% CI 0.85–7.31). Although the risk of malnutrition was higher in SGA using the customized method, the test of interaction between the log of the relative risks was not statistically significant (z-score = − 0.831; *p* = 0.2).

It is important to note that, in the overlap, 33.3% of children classified as SGA according to the customized method and as AGA according to INTERGROWTH21st had an PI below the 10th centile.

Newborns classified as LGA, either both by INTERGROWTH21st or by the customized method, had a greater risk of presenting a PI >90th centile. However, the RR of overnutrition in newborns classified as LGA by the customized method was higher (RR: 5.26, 95% CI: 2.95–9.36) than that of newborns classified as LGA by INTERGROWTH21st (RR 3.57, 95% CI: 1.89–6.74). Again, the comparison of the logarithms of both risks (INTERGROWTH21st and custom method) showed that the differences found between both methods were not statistically significant (z-score = − 0.884; *p* value = 0.19).

It is important to clarify that, in the overlap, 86.2% of children classified as LGA by INTERGROWTH21st and as AGA by the custom method presented a normal IP and only 10.3% presented a PI>90th centile.

Table 4 shows the ability of INTERGROWTH21st and our customized method to identify a PI <10th centile in newborns classified as SGA or a PI >90th centile in newborns classified as LGA. Identifying malnutrition, both PPV and NPV were superior using the customized method than using INTERGROWTH21st.

**Table 4:** Sensitivity, specificity, predictive values and likelihood ratios of the INTERGROWTH21st and customized methods for identification of neonatal malnutrition and overnutrition

	Malnutrition (PI < 10th centile)		Overnutrition (PI > 90th centile)	
	INTERGROWTH21st SGA	Customized method SGA	INTERGROWT21st LGA	Customized method LGA
Sensitivity	15.79% (0–34,82)	40% (16.03–63.97)	59.38% (40.80–77.95)	50% (31.11–68.89)
Specificity	94.24% (90.01–98.48)	89.63% (84.66–94.60)	76.61 (69.97–83.25)	90.74% (85.97–95.51)
PPV	27.27% (0–58,14%)	32% (11.71–52.29)	32.20% (19.43–44.97)	51.61% (32.41–70.82)
NPV	89.12% (34.74–94.49)	92.45 (88.03–96.87)	90.97% (85.94–96.00)	90.18% (85.31–95.06)
LR+	2.74 (0.80–9.45)	3.86 (1.92–7.77)	2.54 (1.71–3.77)	5.40 (2.98–9.78)
LR-	0.89 (0.73–1.09)	0.67 (0.47–0.96)	0.53 (0.35–0.81)	0.55 (0.39–0.78)

*PI* ponderal index, *SGA* Small for gestational age, *LGA* Large for gestational age, *PPV* Positive predictive value, *NPV* Negative predictive value, *LR+* Positive likelihood ratio, *LR-* Negative likelihood ratio

**Fig. 1:**
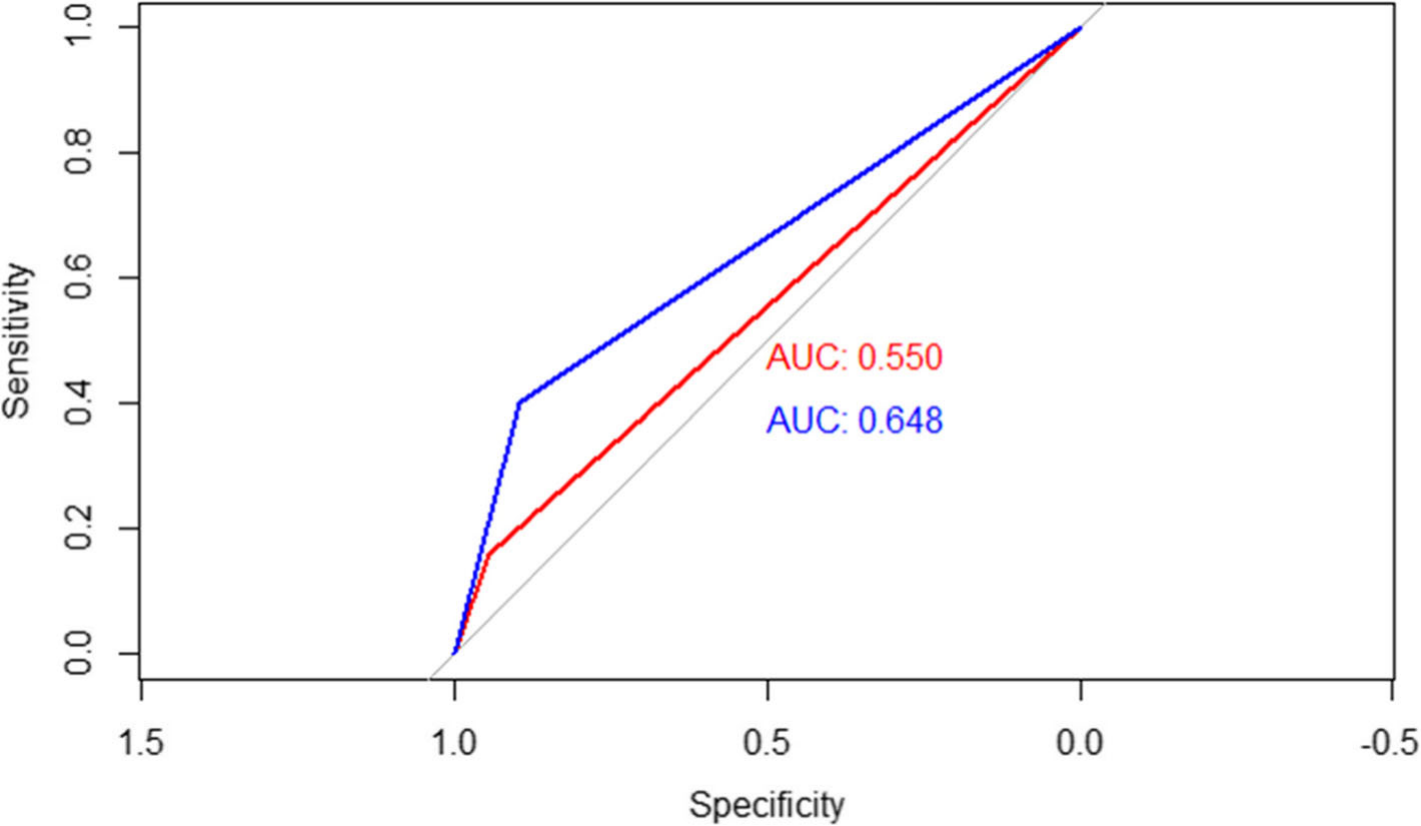
ROC curves of INTERGROWTH21st and customized method in the identification of malnourished newborns. Newborns classified as SGA or AGA have been included for each of the methods, setting the 10th centile as a cut-off point. The test has been considered positive when the birth weight index has been below 10th centile

Figure 1 shows the ROC curves of both methods for the detection of a PI <10th centile. The AUC obtained with INTERGROWTH21st was 0.550 while using the custom method we obtained an AUC of 0.648. Although the customized method obtained a higher AUC, the comparison of both curves using the deLong method showed that the differences found were not statistically significant (*p* = 0.18).

Identifying children with a PI > p90 (overnutrition) the PPV of the customized method to was significantly higher than that of INTERGROWTH21st. For detecting overnutrition, the PPV of the customized method was 51.61% (95% CI 32.41–70.82) versus 32.20% (95% CI 19.43–44.97) for INTERGROWTH21st. However, NPVs of the two methods were very similar.

Identifying overnutrition (PI>90th centile), the LR+ of the customized method was superior for identifying newborns with a PI > p90.

**Fig. 2:**
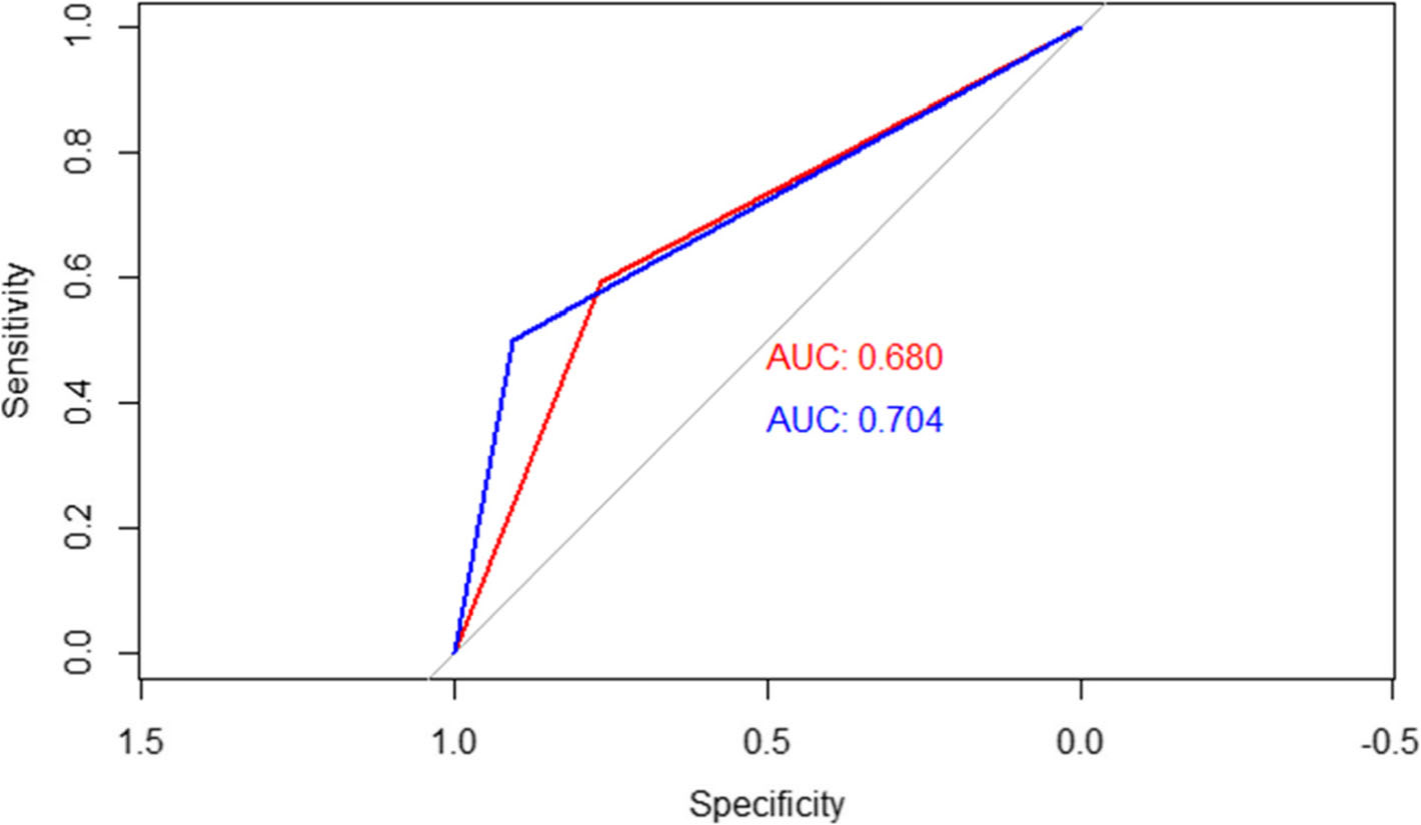
ROC curves of INTERGROWTH21st and customized method in the identification of overnourished newborns. Newborns classified as LGA or AGA have been included for each of the methods, setting the 90th centile as a cut-off point. The test has been considered positive when the birth weight index has been found above 90th centile

Figure 2 shows the ROC curves obtained by both methods in the identification of newborns with a PI >90th centile. The customized method obtained an AUC that was slightly higher than that obtained by INTERGROWTH21st (0.71 vs. 0.68). However, this difference was not statistically significant (*p* = 0.72).

## Discussion

The classification of fetal nutritional status via an appropriate ultrasound standard is important to guide pregnancy management. A fetus incorrectly classified as SGA or LGA will induce the clinician to intensify the monitoring of the pregnant woman and, in the specific case of the GDM, even to modify the diet or treat with insulin. Therefore, we believe that the clinician should choose the curve that best identifies newborns with true alterations in nutritional status (malnutrition or overnutrition).

This study shows that in newborns of mothers with GDM, the rates of SGA and LGA differ by the reference curve used, INTERGROWTH21st or customized. The SGA rate using INTERGROWTH21st was 4.8%, significantly lower than 10.8% observed using customized curves. In contrast, the LGA rate using INTERGROWTH21st was 26%, compared to 13.4% using our customized curves as the reference. Therefore, in our population, the customized method identifies more SGA while INTERGROWTH21st identifies more LGA.

These SGA results were consistent with those recently published by Francis et al. [15] who reported overall SGA and LGA rates of 10.5 and 9.5%, respectively, using customized curves. Using INTERGROWTH21st Francis et al. observed an overall SGA rate, 4.4%, very similar to the 4.7% rate of our sample and, like our study, they find an unexpectedly high LGA rate of 20% (Similar to our 25%, if we take into account that we have analyzed a diabetic population).

Similarly, Anderson et al. [21], reported a significantly lower SGA rates using INTERGROWTH21st versus customized curves (4.5% vs 11.6%); additionally, in their cohort, Anderson et al. had a customised LGA rate of 8.9% and INTERGROWTH21st LGA rate of 20.8%, with wide variation by ethnicity (European women 23.7%, Indian 6.8% and Pacific 32.%) [NH Anderson, personal communication].

The use of PI to assess the nutritional status of the newborn presents certain limitations as it not only evaluates fat mass, but also head size, lean mass and bone mass, hence potentially limiting its accuracy in reflecting adiposity. However, accurate measures of body composition are usually costly. A recent work published by Chen et al. [33] informs us that although skinfold measures may have more discriminative power in terms of total body adiposity, simple anthropometric measures (like PI) correlated strongly with neonatal adiposity and conclude that these simple measures could be of value in large epidemiological studies.

We found that the SGA and LGA classifications by each method (customized vs INTERGROWTH21st) reflect differences in their ability to identify true alterations in the PI as an indicator of the neonatal nutritional status.

The RR of malnutrition (PI <10th centile) in newborns classified as SGA by customized curves was higher, than that of newborns classified as SGA by INTERGROWTH21st. This may be since 33.3% of children classified as SGA by the customized method, but as AGA by INTERGROWTH21st, had a PI <10th centile (suggestive of malnutrition). In any case, when we compare both RR, we did not find a statistically significant difference (*p* = 0.2).

Likewise, the accuracy of the customized curves for identification of newbornt with a PI <10th centile was greater than that of INTERGROWTH21st, LR + of 3.86 vs 2.74, respectively. That is, using customized curves, it is 3.86 times more likely that a malnourished newborn is classified as SGA than a normally nourished newborn is classified as SGA.

In a previous study by our team [22], carried out in an unselected population, the customized method was superior to the population-based for the identification of newborns with a PI at birth <10th centile. This superiority of the customized method was more evident in the highest scales of maternal weight and height.

Owen et al. [34] found a similar relationship between customized birth weight percentiles and neonatal malnutrition, but concluded that, in a low-risk population, the customized curves are only moderately useful in the identification of neonates with a low PI, with a positive likelihood ratio of 4.3 (95% CI: 2.5–7.1). Agarwal et al. [35] also found that the PI at birth was lower in newborns classified as SGA by customized curves than in SGA according to population curves. The apparent superiority of the customized method to detect a PI <10th centile should be interpreted with caution since the difference found between the RR was not statistically significant and when comparing the ROC curves of both methods the difference found was not statistically significant. The reality is that, with the prevalence of malnutrition found in the sample (8.9%) we would need at least 1200 individuals to reach a statistical power of 80%. This shows that the statistical non-significance could be due to an insufficient sample size.

Similarly, the RR of overnutrition (PI>90th centile) associated with LGA classification by customized curves, RR 5.26, was greater than in the newborns classified as LGA by INTERGROWTH21st, RR 3.57). It should be noted that this result was obtained despite the fact that the proportion of children classified as LGA by INTERGROWTH21st was significantly higher than using the customized method (25.6% vs. 13.2%) and it is explained why the majority (86.2%) of LGA children according to IG but AGA according to the custom method presented a normal PI. This difference should also be interpreted with caution since the RRs found showed wide confidence intervals and since, the subsequent comparison of both RRs showed that the differences found were not statistically significant (*p* = 0.19).

Further, our analysis of the accuracy of each method for identification of newborns with a PI > p90 revealed that the customized method had a greater LR+, 5.40, than the LR+, 2.54, using INTERGROWTH21st. Hence, using customized curves, it is 5.40 times more likely that an over nourished newborn will be classified as LGA than a normally nourished newborn will be classified as LGA. Given that in GDM it is critical to identify fetal overnutrition, we consider of special relevance the differences found in the PPV of both methods to identify a PI > p90. Using our customized curves, the probability that a fetus classified as LGA has a PI >90th centile is 51.61% while using INTERGROWTH21st the probability drops to 32.20%. These results are consistent with those found by Gonzalez et al. [36] However, in our study, an analysis of the ROC curves shows that the AUC obtained by both methods is very similar and the small difference observed (0.70 vs. 0.68) is not statistically significant. Again, it should be noted that, with the prevalence of newborns with PI>90th centile, the lack of significance could be due to an insufficient sample size since 1603 individuals would have been required to have a statistical power of 80%.

Another aspect worth discussing is the low sensitivity of both methods to identify newborns under-nourished in fetuses classified as SGA. However, in the same case, the specificity is acceptable. In our opinion, this shows that the same cut-off point (10th centile for SGA) can classify a child as normal or small depending on the reference curve.

The relatively small sample lead to our primary limitations, including occasional RRs with overlapping or wide confidence intervals, which hampered their interpretation. However, the relative risks were usually large enough to be taken clinically relevant. It should be noted that the premises from which we started when estimating the sample size have not been fulfilled. In our estimation, we assumed a ratio between non-exposed / exposed of 8. This was based on the premise that we would find approximately 10% SGA and 10% LGA with each method. However, using INTERGROWTH21st, for example, we found 163 AGA and only 11 SGA. This makes the non-exposed / exposed ratio rise to 14.8. With this ratio, we would have needed 488 AGA newborns to obtain significance (and this to detect a minimum risk of 3; to detect a minimum risk of 2.5 - which is the observed one - we would need an even larger sample: 51 SGA and 751 AGA). Therefore, although in general our study seems to indicate that the customized method could surpass INTERGROWTH21st in the identification of alterations in nutritional status, we think that it is necessary to complement this study with a larger sample.

In addition, selection and information biases could affect the estimated of the performance of the two reference curves. We believe that our results can be extrapolated to other populations of pregnant women with adequate monitoring because obstetricians, endocrinologists, family doctors and primary care midwives monitored the pregnant woman with GDM using criteria for diagnosis, follow-up and treatment established by the Spanish Society of Gynecology and Obstetrics.

## Conclusions

Overall, there does seem to be a difference in performance between the method customized and INTERGROWTH21st. Our results show that customized LGA and SGA may be better identifying nutritional status as assessed by PI in diabetic mothers. However, further studies with a larger sample size are necessary to increase the reliability of these findings.

In our opinion, if our results are confirmed, the greater capacity of the customized curves to identify newborns with PI > p90 may have important implication for monitoring pregnant women with GDM because intrauterine identification of overnutrition may indicate poor maternal metabolic control and the need for extreme dietary care or, even, insulin treatment.

## Data Availability

The datasets generated and/or analysed during the current study are available in ONEDRIVE with this link https://1drv.ms/u/s!AnSC-9_Msj6-g4gXADvN6r9fwAfQ8w?e=YlUY3e

## Abbreviations

AGA: Adequate for gestational age
CI: Confidence interval
EFW: Estimated fetal weight
GDM: Gestational diabetes mellitus
LGA: Large for gestational age
LR-: Negative likelihood ratio
LR+: Positive likelihood ratio
NPV: Negative predictive value
PI: Ponderal index
PPV: Positive predictive value
RR: Relative risk
SGA: Small for gestational age
